# Challenges with misclassification of American Indian/Alaska Native race and Hispanic ethnicity on death records in North Carolina occupational fatalities surveillance

**DOI:** 10.3389/fepid.2022.878309

**Published:** 2022-10-21

**Authors:** Elizabeth S. McClure, Danielle R. Gartner, Ronny A. Bell, Theresa H. Cruz, Maryalice Nocera, Stephen W. Marshall, David B. Richardson

**Affiliations:** ^1^NC Occupational Safety and Health Education and Research Center, Gillings School of Global Public Health, University of North Carolina at Chapel Hill, Chapel Hill, NC, United States; ^2^Department of Epidemiology & Biostatistics, College of Human Medicine, Michigan State University, East Lansing, MI, United States; ^3^Division of Public Health Sciences, Department of Social Sciences and Health Policy, Wake Forest School of Medicine, Winston-Salem, NC, United States; ^4^Office of Cancer Health Equity, Wake Forest Baptist Comprehensive Cancer Center, Winston-Salem, NC, United States; ^5^North Carolina American Indian Health Board, Winston-Salem, NC, United States; ^6^Department of Pediatrics, University of New Mexico, Albuquerque, NM, United States; ^7^UNM Prevention Research Center, Albuquerque, NM, United States; ^8^University of North Carolina Injury Prevention Research Center, Chapel Hill, NC, United States; ^9^Department of Epidemiology, Gillings School of Global Public Health, University of North Carolina at Chapel Hill, Chapel Hill, NC, United States; ^10^Environmental and Occupational Health, Program in Public Health, University of California, Irvine, Irvine, CA, United States

**Keywords:** disparities, race, ethnicity, occupational epidemiology, mortality studies

## Abstract

As frequently segregated and exploitative environments, workplaces are important sites in driving health and mortality disparities by race and ethnicity. Because many worksites are federally regulated, US workplaces also offer opportunities for effectively intervening to mitigate these disparities. Development of policies for worker safety and equity should be informed by evidence, including results from research studies that use death records and other sources of administrative data. North Carolina has a long history of Black/white disparities in work-related mortality and evidence of such disparities is emerging in Hispanic and American Indian/Alaska Native (AI/AN) worker populations. The size of Hispanic and AI/AN worker populations have increased in North Carolina over the last decade, and North Carolina has the largest AI/AN population in the eastern US. Previous research indicates that misidentification of Hispanic and AI/AN identities on death records can lead to underestimation of race/ethnicity-specific mortality rates. In this commentary, we describe problems and complexities involved in determining AI/AN and Hispanic identities from North Carolina death records. We provide specific examples of misidentification that are likely introducing bias to occupational mortality disparity documentation, and offer recommendations for improved data collection, analysis, and interpretation. Our primary recommendation is to build and maintain relationships with local community leadership, so that improvements in the ascertainment of race and ethnicity are grounded in the lived experience of workers from communities of color.

Workplaces are important determinants of racial and ethnic disparities in diseases and mortality. They can also be environments of exploitation, intimidation, and racism. Segregation of employment is common, with non-white workers overrepresented in dangerous and low paying jobs (1), and racial exploitation is common within jobs where non-white workers are employed. The COVID-19 pandemic has highlighted the significant role of work settings in driving health disparities (2). In North Carolina, the occupational fatality rate in the male workforce is 8 deaths per 100,000 worker years, with rates among Black, Hispanic, and American Indian or Alaska Native (AI/AN) workers being 1.5–3 times the rates in the non-Hispanic white workforce (3, 4). However, workplaces constitute more readily regulated environments than many other drivers of health disparities and therefore provide opportunities for exposure prevention (5), and work-related deaths are often preventable. Therefore, workplaces are essential sites for public health interventions aimed at reducing health disparities.

We recognize that race and ethnicity are social constructs with complex cultural and political influences, and therefore do not have “correct” classifications. We also acknowledge that consistency between self-identification and death record documentation of race and ethnicity is important for informing measures to address workplace and health inequities. While there are many relevant indicators to consider, mortality studies are a mainstay of epidemiological research on occupational injury and disease, and much of occupational injury and disease surveillance also draws upon information collected from death certificates (6). Additionally, legal advocacy relies on accurate reporting of protected racial and ethnic identities in death records to substantiate reports and litigation to hold employers accountable for worker safety (7). To facilitate such research, contemporary death certificates record usual occupation and industry of the decedent as well as an indication of injury at work. However, there are notable obstacles to using death certificates to investigate racial and ethnic disparities in occupational injury and disease (8).

In the United States (US) South, Black men experience the highest rate of fatalities due to occupational injury, and the rate among Hispanic men is increasing (4, 9), but misclassification of decedents' race and ethnicity is likely resulting in underestimation of these disparities. The vast majority of occupational fatalities occur in the male workforce, but racial and ethnic disparities persist in rates among female workers (9). State and federal government agencies can regulate workplace safety with structural controls, like engineering improvements and individual controls, like protective equipment (10). Regulations to mitigate disparities rely on accurate and consistent characterization of populations to understand the workplace hazards and safety experienced by different groups as well as the true disparities in mortality by group. While North Carolina (NC) has the largest AI/AN population in the eastern US, and NC's Asian, AI/AN, Hispanic, and “two or more race” Census-defined racial/ethnic groups have grown in the past 10 years, these groups represent a smaller proportion of the population than many other states (11). Previous research on racial and ethnic mortality disparities suggests that states with smaller non-white populations tend to have more incongruence between individuals' self-identified and death certificate recorded race and ethnicity (12–18). Further, a small number of misclassifications among small populations can have big impacts on mortality statistics (19).

This commentary examines obstacles related to classification of decedents with respect to race and ethnicity. In this essay, we describe problems in death record race and ethnicity classification and their implications for NC public health efforts. We then offer models for better race and ethnicity classification among decedents, particularly for AI/AN and Hispanic decedents. Throughout, we will be using the terms “American Indian/Alaska Native (AI/AN)” and “Hispanic,” to be consistent with US Census Bureau and other administrative data sources. We acknowledge that these terms have origins in colonizer logic, oversimplify heterogeneous groups, and are proxy reflections, at best, of the diverse and dynamic nature of individuals' racial and cultural identities. A list of key terms and their definitions is provided in Table 1.

**Table 1:** Definition of key terms as used in the manuscript.

**Term**	**Definition**
Blood quantum	A colonizer-created, metaphorical quantification of an individual's proportion of “Indian blood” implemented to limit tribal membership (20–23).
Ethnicity	The unique socio-cultural characteristics of groups.
Federally recognized Tribes	Tribes recognized by the US government and eligible for services from the Bureau of Indian Affairs. In North Carolina, only the Eastern Band of Cherokee Indians is a federally recognized Tribe (24).
Hispanic ethnicity categories, NC death certificates	No, not Spanish/Hispanic/Latino; Yes, Mexican, Mexican American, Chicano; Yes, Puerto Rican; Yes, Cuban; and Yes, other Spanish/Hispanic/Latino (25).
Hispanic ethnicity categories, US Census	Not Hispanic or Latino; Mexican, Mexican American, Chicano; Puerto Rican; Cuban; and “Another Hispanic, Latino, or Spanish origin (11).
Indigenous	The original inhabitants of a territory that has been colonized by a foreign population (26).
Race	A social construct that identifies one's location in the social hierarchy based primarily on skin color (27).
Racial categories, NC death certificates	White; Black or African American; American Indian or Alaska Native (including name of enrolled or principal tribe); Asian Indian; Chinese; Filipino; Japanese; Korean; Vietnamese; Other Asian (Specify); Native Hawaiian; Guamanian or Chamorro; Samoan; Other Pacific Islander (Specify); and Other (Specify) (25).
Racial categories, US Census	Individuals who identify as White, Black or African American, American Indian or Alaska Native, Asian, Native Hawaiian or Other Pacific Islander, or Some other race (11).
Racial or ethnic health disparity	A health difference among groups of people who have systematically experienced greater obstacles to health based on their racial or ethnic group (28).
State recognized Tribes	Tribes recognized by state government. In North Carolina these include the Coharie Tribe, Eastern Band of the Cherokee Indians, Haliwa-Saponi Indian Tribe, Lumbee Tribe of North Carolina, Meherrin Nation, Occaneechi Band of the Saponi Nation, Sappony, and Waccamaw Siouan Tribe (29).

## Current practices for recording and analyzing race and ethnicity among North Carolina occupational fatalities

In the US, each state has its own regulations for death certificate registration and completion, and the National Center for Health Statistics provides guidance to encourage data consistency within the National Vital Statistics System in the form of a *Funeral Director's Handbook* (30). In NC, information on race and ethnicity of decedents is routinely recorded on the death certificate. Race and ethnicity are also recorded in the medical examiner's record, in addition to the death certificate, if the death is investigated by the medical examiner. The investigated deaths are those due to homicide, suicide, unintentional injury, trauma, disaster, violence, unnatural, unknown, or suspicious circumstances (31–33). The investigating medical examiner also reports whether the death, or injury resulting in death, occurred at work. The medical examiner's assessment of race and ethnicity is typically based upon information from the funeral director, who is expected to request the information from next of kin or other informants. In addition, the medical examiner is required to examine the decedent body, and this may further influence their perceptions (4). The race and ethnicity as perceived by the funeral director is recorded on the death certificate, while the perception of the medical examiner will be recorded on the medical examiner's record, or be entered from the death certificate itself. Regardless, on the death certificate, one may select from a list of options for designation of ethnicity, indicating “Hispanic origin” including: No, not Spanish/Hispanic/Latino; Yes, Mexican, Mexican American, Chicano; Yes, Puerto Rican; Yes, Cuban; and Yes, other Spanish/Hispanic/Latino. They may select one or more races including: White; Black or African American; American Indian or Alaska Native (including name of enrolled or principal tribe); Asian Indian; Chinese; Filipino; Japanese; Korean; Vietnamese; Other Asian (Specify); Native Hawaiian; Guamanian or Chamorro; Samoan; Other Pacific Islander (Specify); and Other (Specify). These race and ethnicity fields are standard across states as of 1997 but have changed over time. They are recommended by the National Center for Health Statistics and can be further aggregated into the minimum five racial categories required by the Office of Management and Budget (OMB) (30).

Since 1987, all states in the US contribute to the Census of Fatal Occupational Injuries (34). The Trends and Disparities in Fatal Occupational Injuries in North Carolina Research Team (herein referred to as “Team”)—a partnership between the University of North Carolina (UNC) Injury Prevention Research Center, the UNC Department of Epidemiology, the North Carolina Department of Health and Human Services (NC DHHS), the NC Office of the Chief Medical Examiner, and the Mecklenburg County Medical Examiner's Office—receive full narrative reports of each occupational injury-related death for review and analysis. The narrative reports can include the death certificate, autopsy reports, and any related news articles (3). The Team analyzes the data and prepares reports for scientific publications, NC DHHS, and the National Institute for Occupational Safety and Health (NIOSH).

**Figure 1:**
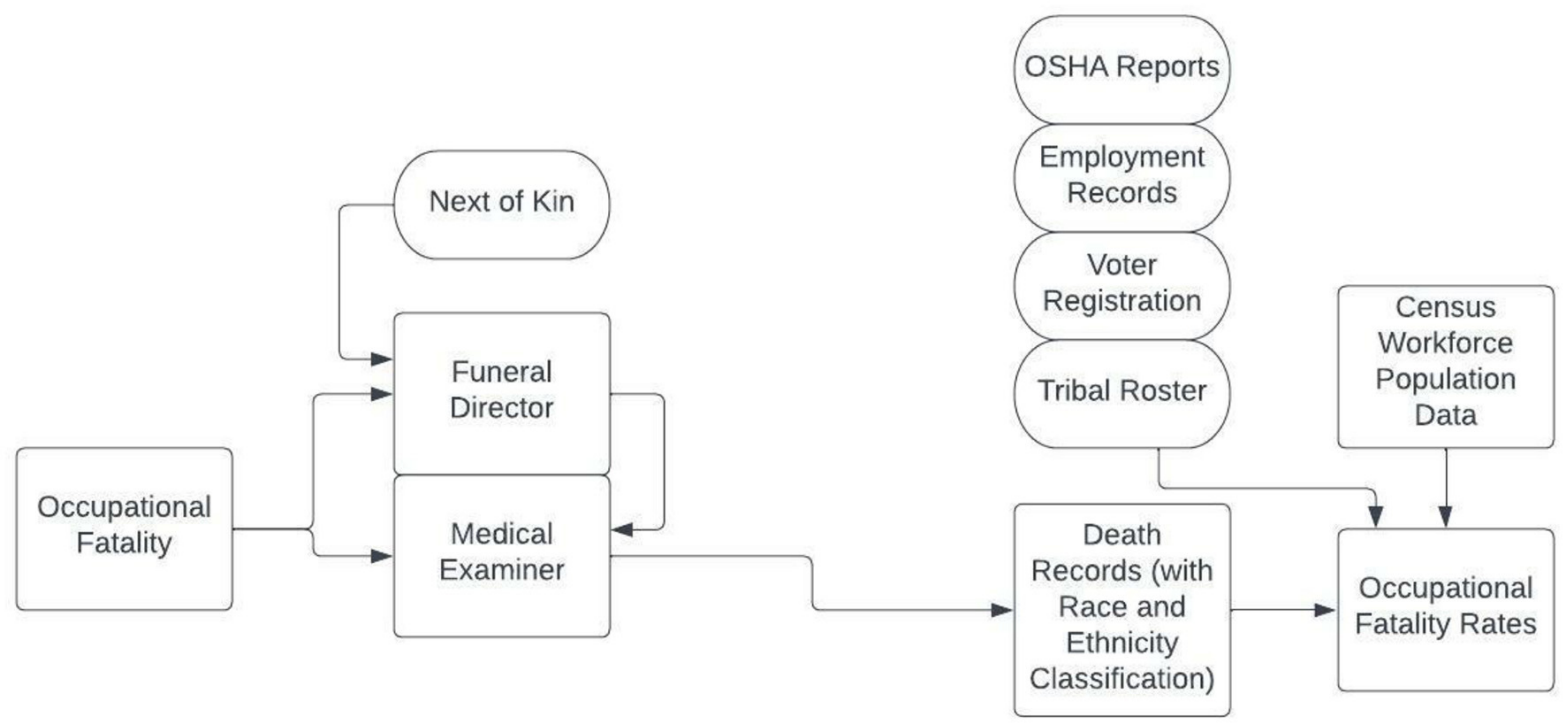
Flow chart of race and ethnicity classification in North Carolina occupational fatality rates.

Figure 1 shows the process of data collection and reporting for occupational fatalities in NC. Not all decedents interact with a funeral director, but all occupational fatalities are required to be reviewed by the medical examiner. If a funeral director is involved, the medical examiner will use the data collected by the funeral director to complete the death certificate. Race and ethnicity information may come from administrative sources of self-reported data (e.g., employment records, voter registration, or tribal rosters). When self-reported data is not available or accessed, funeral directors are expected to collect race and ethnicity data from next of kin. Occupational fatality rates are calculated using the number of fatalities divided by the number of workers in the population, as collected and reported by the US Census. These rates are typically multiplied by a constant (e.g., 100,000) for ease of reporting. The US Census collects self-reported race and ethnicity data, though occasionally rely on neighbor report of this information for nonresponding households. Although the ideal would be to use self-reported race and ethnicity for both the numerator and denominator, the reality is that numerator data isn't always collected in this way. Therefore, the classification issue is twofold: some self-identifying AIAN and Hispanic people are not identified as such on their death certificates, and there is incongruence in data collection processes/sources for race and ethnicity information for numerators and denominators.

## Problems in decedent race and ethnicity classification and disparities documentation

Throughout the US, information on race and ethnicity recorded on the death certificate is less accurate for non-white populations than for whites, a disparity partly due to inadequate training (12–18). Kalweit et al. conducted surveys and interviews with funeral directors across the US. They found that there is inconsistent training for funeral directors, and funeral directors do not follow standard methods for collecting and coding “Hispanic origin” and tribal affiliation on death certificates (18). NC funeral directors shared in informal interviews that they frequently report their own perceptions of the decedent's race without consulting next of kin. This is typically concordant between funeral directors and next of kin for Black and white decedents, because of segregated funeral home patronage. However, ethnicity, multiple race, and smaller race group affiliations are more often misidentified. This is consistent with research on the misclassification of race and ethnicity based on geocoding and surname analyses which has shown better accuracy among more segregated populations and in areas where there are higher concentrations of a racial or ethnic population (35). Barriers to accurate race and ethnicity coding for decedents from AI/AN and Hispanic populations are especially salient in NC, where AI/AN and Hispanic workers are overrepresented in dangerous workforces (4, 32), have heterogeneous identities, and experience varied racialization and political disempowerment (36–38).

### Theoretical issues with American Indian/Alaska Native race classification

A broad issue with classifying AI/AN as a racialized identity is that this characterization is rooted in colonizer logic that perpetuates racist science. Other dimensions of AI/AN group membership are important to consider, particularly political identity as citizens of sovereign nations. This political identity is unique in that no other racialized group in the US holds this status. There can be incongruence between a racialized identity and a political identity. The political identity, by way of imposition of federal policies of recognition and resource allocation, often reifies (erroneously makes concrete) the racial logic. Using colonizer logic, one can be “a little bit” Native American. Using citizenship-based membership metrics, one cannot be part citizen of a tribe, just like no one is “a little bit” of a US citizen. Each native nation has the right to determine its criteria for citizenship and there is heterogeneity across Indian Country in how this is done (e.g., lineal descent or blood quantum requirements—a colonizer-created, metaphorical quantification of individuals' proportion of “Indian blood” implemented to limit tribal membership) (20–23). For example, members of the Lumbee Tribe of North Carolina, enroll and reregister at the enrollment office in Pembroke, NC using genealogy charts linking their lineage to specific names on the 1900 and 1910 Census (39).

Who gets categorized into what group, using whose definition, has implications for what research questions can be answered about who or whose health status is recorded and using what methods. Further complicating use of health surveillance data, are the metrics of belonging or self-identification for AI/AN people. Adoption, marriage, cultural practices, language use, and geographic proximity to native communities are all used locally to define group membership (21, 22, 39). NC has one federally recognized tribe (the Eastern Band of Cherokee Indians) and seven state recognized tribes (Coharie Tribe, Haliwa-Saponi Indian Tribe, Lumbee Tribe of North Carolina, Meherrin Nation, Occaneechi Band of the Saponi Nation, Sappony, Waccamaw Siouan Tribe). There are also people who identify with tribes that are neither state nor federally recognized. There are large size differences between tribes in NC and many tribal members have moved to urban communities across the state. This has led to the establishment of urban Indian Centers in Charlotte, Greensboro, Raleigh and Fayetteville (29). NC is also home to citizens of tribes with homelands outside of NC.

### Theoretical issues with Hispanic ethnicity classification

The term “Hispanic” can include anyone of Spanish descent and refers to linguistic origins, but individuals who do not speak Spanish may still identify as Hispanic. The terms “Latino/a/x” refer to individuals with origins in Latin America and the Caribbean, including French-speaking Haitians and Portuguese-speaking Brazilians (40). Both terms have imperial histories and related cultural implications (for example, someone with Portuguese ancestry may be strongly opposed to affiliating with Spanish descent) (38, 40). The most used term, “Hispanic,” is outdated and unlikely to reflect the internalized ethnic identity of many members of NC's communities (37). The term emphasizes Spain's colonial power and ignores the cultural influences of Central and Latin American countries and populations. “Hispanic” is unlikely to resonate with a person living in North Carolina who sees themselves as Guatemalan, for example. Hispanic identifying individuals have varied and overlapping ancestries, racial identities, and perceived races (38). In health surveillance and research, inconsistencies between population definitions and names has created barriers to specific intervention design and targeting (41). For consistency with Census and CDC data sources, we use “Hispanic” in this commentary.

### Issues with race and ethnicity data collection on the death certificate

Funeral directors or clinicians filling out death certificates are ascribing race using largely unknown criteria that are likely heterogeneous. Anecdotal discussions with NC funeral directors suggest that AI/AN identities are only recorded if there is an obvious indication of tribal membership. Depending on a host of factors, this may result in many AI/AN individuals getting misidentified as white and/or Hispanic on their death certificate. Racial classification by the funeral director or medical examiner is often subjective or based on physical appearance (skin color, hair texture, facial features) or other similar cues of the decedent (clothing upon arrival, tattoos, conversations with family), including racial stereotyping. Additionally, the criteria provided in funeral director guidance may be ambiguous with respect to who qualifies as AI/AN. AI/AN individuals with Hispanic sounding surnames might be classified as Hispanic and white (42). In the US, misclassification of a decedent's race and ethnicity is most common for AI/AN people (25). AI/AN populations are often undercounted or aggregated into “other” categories in administrative data because they are one of the smallest recognized racial/ethnic groups in the US, and individuals from Indigenous communities are likely to identify with multiple racial/ethnic groups (36). Combining heterogeneous groups in the context of mortality data can hide disparities (19, 43). The increases in NC's “two or more race” group population indicates we risk masking disparities in this way, which may obscure problems of relatively high mortality rates in AI/AN worker groups.

Hispanic ethnicity classification is also frequently based on the funeral director's knowledge of the family or personal observations (44), which is influenced by stereotypes of names, physical appearances, and use of Spanish language by family members. Conflation of race and ethnicity is also common. Funeral directors often include indicators of Hispanic ethnicity in the “other description” field for race on the death certificate and leave the ethnicity field blank (17, 32). Funeral directors in NC indicated that they typically record Hispanic ethnicity information if the family speaks Spanish, or if the name sounds Spanish. Several death records included in the Trends and Disparities in Fatal Occupational Injuries in North Carolina data included birth places in South and Central American countries among decedents not classified as Hispanic. It is also common to leave the race field blank for decedents who are identified as Hispanic (17). The Funeral Director's Handbook recommends choosing only one category in the ethnicity field before acknowledging that some decedents may identify with multiple Hispanic ethnicities (30). Decedents identifying with multiple ethnicities are subject to similar issues as those identifying with multiple race groups, likely leading to underestimated disparities. Further, concretizing these stereotypes into vital statistics data contributes to reification of race and ethnicity by pathologizing membership in a minority racial or ethnic group (45, 46). For example, Noymer et al. find that individuals' coded cause of death impacts the race and ethnicity classification in death records. Specifically, individuals whose deaths are due to cirrhosis are more likely to be coded as AI/AN, and homicide decedents are more likely to be coded as Black (47). This facilitates a narrative of biological determinism for mortality disparities that are, in reality, shaped by economic and social structures.

Another challenge is the lack of quality assurance. Data may be inaccurate due to data entry errors, providers may not collect race and/or ethnicity information (and this missingness may be differential by race and ethnicity), or there may be a lack of follow up with next of kin in cases where racial identity is unclear. There is also variation in misidentification of AI/AN people by age [children and elderly decedents are misidentified more often as white (17, 48)], cause of death (17, 47), residential location (urban vs. rural) (17, 48, 49), physical appearance, and blood quantum [individuals with lower blood quantum are misclassified as white in clinical settings (48)]. For Hispanic decedents, there is variation in misclassification by nativity (foreign born decedents are more likely to be accurately identified than US-born decedents), race (non-white decedents are more likely to be misclassified as non-Hispanic than white or “other” race decedents) (17), Hispanic subgroup (individuals who are not Mexican or Chicano are more likely to be misclassified as non-Hispanic) (12, 16, 17), region of residence, and degree of co-ethnic concentration (decedents who lived in a location with a lesser degree of co-ethnic concentration at the time of death are more likely to be misclassified) (16, 17).

**Figure 2:**
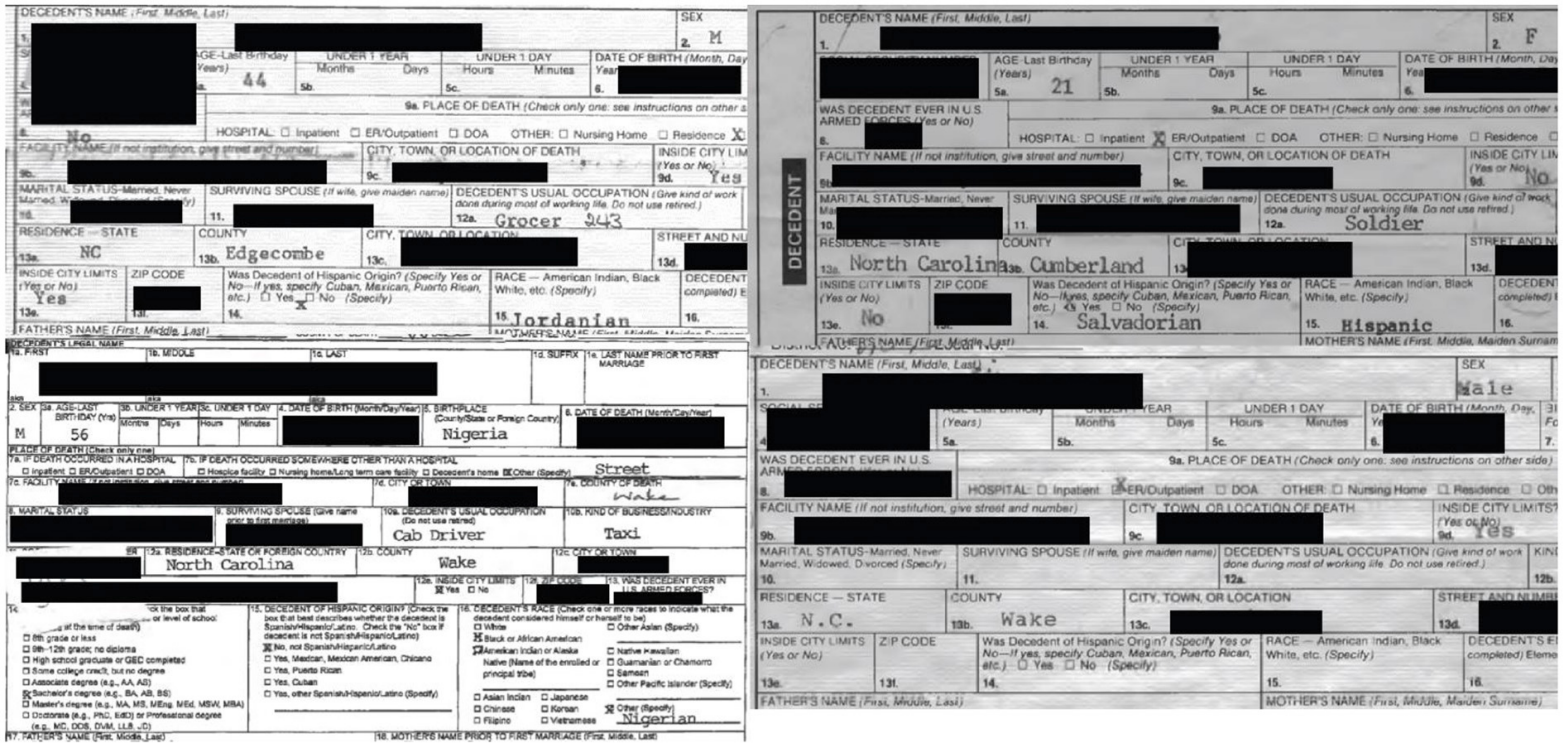
Examples of common race and ethnicity misclassification on North Carolina death certificates of occupational fatality decedents.

We provide four examples of common issues in race and ethnicity classification on NC death certificates in Figure 2. These represent the observable subset of issues described above (e.g. conflation of ancestral origin with race or ethnicity). The race and ethnicity prompt wording and format varies slightly across years of death certificate forms, and the figure includes forms from the 1990's, 2000's, and 2010's. In the top left example, the decedent's ethnicity is indicated as not Hispanic, and “Jordanian” is entered in the race field. The top right example has “Salvadorian” entered into the ethnicity field and “Hispanic” in the race field. In the bottom left example, “no” is indicated in the Hispanic ethnicity field, and both “Black or African American” and “AI/AN” races are indicated, while the free text “Nigeria” is also entered in the race field. Finally, the bottom right example is missing any information on ethnicity and race. In a comparison of 2,170 death certificates and medical examiner records (a 5% sample of the occupational fatality records), 15 records had discrepancies between the hard copy death certificate and the medical examiner's electronic file in the ethnicity field, and 60 records had discrepancies in the race field. This reflects a subset of decedents with misidentified race and ethnicity, as we were unable to validate either data source with reports from community members or next of kin. Further, while these discrepancies are small in absolute numbers of people, they reflect large proportions of individuals in oppressed and overlooked populations. Repeated “small” errors like these amount to erasure of communities.

### Issues with using race/ethnicity death record data in research and surveillance

Researchers (including our team at the Trends and Disparities in Fatal Occupational Injuries in North Carolina project) and public health practitioners often analyze mortality data by calculating risks or rates, in which the numerator of the measure represents the number of deaths, and the denominator represents the number of persons (or person-years) at risk. Population Census data often serve as the basis for denominator estimates of mortality risk and rate calculations, and Census data likely misestimate the size of AI/AN and Hispanic groups (50, 51). For AI/AN groups, there are several factors at play. On the one hand, there are people that, over their life course, discover that they have Native ancestry, potentially leading to overcounting. On the other hand, AI/AN individuals are missed by the Census by up to 11%, and these omissions are more common among younger and male respondents (52, 53). Errors in estimation of denominators result in errors in estimation of AI/AN and Hispanic occupational fatality rates. Group-specific mortality rate estimates often force those self-identifying as multiracial into a single race category. AI/AN individuals are more likely to be multiracial so any bias coded into this process will proportionately impact AI/AN more than other racial groups. This bridging most frequently results in an overestimate of Hispanic identifying AI/AN in Census denominators, which leads to underestimated rates. Further, individual racial and ethnic identities, ways of defining Indigeneity, tribal enrollment status, all vary over time and contexts, as have Census data collection methods (51, 54, 55). New Census questions can exacerbate conflation of ancestral origin and race in the broad, US social dialogue. As a result, individuals who did not experience social or material marginalization are more likely being counted in the denominators of marginalized groups in population, health, and mortality data than they were previously (45, 47, 51). These misclassification issues and underestimated disparities impact the design and distribution of public health interventions aimed at mitigating disparities.

Another common practice in epidemiology that can bias health disparities data is the suppression of data from small populations. Mortality rates, particularly high mortality rates, from AI/AN and Hispanic populations are characterized as “unstable,” due to small numbers and wide confidence intervals. To address this variability, it is common practice to aggregate numerator counts over extended periods of time. These representations can be misleading, as identities and environments can change. With regard to measures of uncertainty, the statistical foundation for estimation of confidence intervals is based on experimental designs in samples, which may not be relevant in contexts where the entire population is represented in a rate. In these instances where we know the “universe” of decedents, wide confidence intervals may falsely connote uncertainty in the actual number of occupational deaths reported in a small population (56).

## Improving decedent race and ethnicity classification

Accurately quantifying birth and death rates is a fundamental task for any state's public health system. Our primary recommendation for providers classifying race and ethnicity of occupational injury decedents is to build and maintain relationships with local community leadership (57, 58). Decedents' community members can help improve accuracy of this complex and nuanced demographic data, and, more importantly, providers can inform tribes and communities of member deaths (59). More AI/AN and Hispanic public health professionals, statisticians, funeral directors, clinicians, etc. should be hired and brought into the process. Increasing representation of AI/AN and Hispanic groups in the workforce is an important strategy for improving data accuracy. This process should be conducted with critical evaluation and awareness of common pitfalls of recent diversity, equity, and inclusion efforts in workplaces that have tokenized and burdened Indigenous and People of Color with educating their white colleagues (60, 61).

Secondarily, NC state health institutions should develop improved data collection and analysis processes, and lead training efforts to improve data quality. Training without relationship building and changes to the workforce is not sufficient for improving consistency of race and ethnicity data. Adequate training in data collection and community engagement must be provided to public health professionals, funeral directors, and medical examiners. Trainings should support the best practices and center the knowledge of Hispanic, AI/AN, and other non-white funeral directors and medical professionals (18). Novel data collection tools could also be used, like demographics surveys completed by next of kin. Trainings should also expand awareness of and linkage with broad data sources, like electronic health registries and tribal records (19, 62). However, mortality and cancer registry linkages with Indian Health Service (IHS) patient records are not perfect and can result in non-Native individuals being misclassified as Native, or Native people being missed who do not use, access, or have the ability to attend IHS facilities (63–66). In NC, where most tribes are not federally recognized and some are not state recognized, access and use of these rosters are also likely to present new challenges.

Best practices for classifying race and ethnicity are to use self-reported data sources (e.g., employment records, tribal rosters, voter registries), and “best guesses” based on assumptions are not appropriate. When self-report is unavailable, race and ethnicity data should be obtained from next of kin (13, 18, 25, 47). Death record data should allow decedents to have multiple race identities, in order to avoid masking disparities through aggregation of heterogeneous groups. When group-specific rates are calculated, individuals with two or more racial or ethnic identities should be counted in each group with which they identify, and AI/AN individuals' political identity, or formal enrollment and connection to Native Nations, should be privileged (67).

Validation studies (68) can both serve to identify problems in classification of decedents (and therefore inform interventions to improve death records) and serve as a basis to inform methods for “correction” of bias in rate estimates derived from death records that suffer imperfect sensitivity and specificity of classification of decedents with respect to race or ethnicity (69). Boyd et al. recently proposed a new standard for publishing research documenting health inequities, and many of these principles can be applied to mortality data collection (70). By clearly defining and describing race and ethnicity constructs, documentation of mortality disparities can help elucidate differences between ancestry, race, and ethnicity. Further, researchers and government agencies can highlight occupational sources of racial and ethnic mortality disparities, countering the increased popular interest in genetic origins of disease and resisting narratives that pathologize non-white racial and ethnic groups (71). The state medical examiner system has been a useful tool for surveillance of occupational fatalities in NC. Research using this system involves review of documents by a team of researchers and public health practitioners, which currently includes no AI/AN or Hispanic identifying individuals. This is an opportunity to improve the collection, classification, analysis, and results sharing processes for data on race and ethnicity disparities in occupational fatalities in NC.

**Figure 3:**
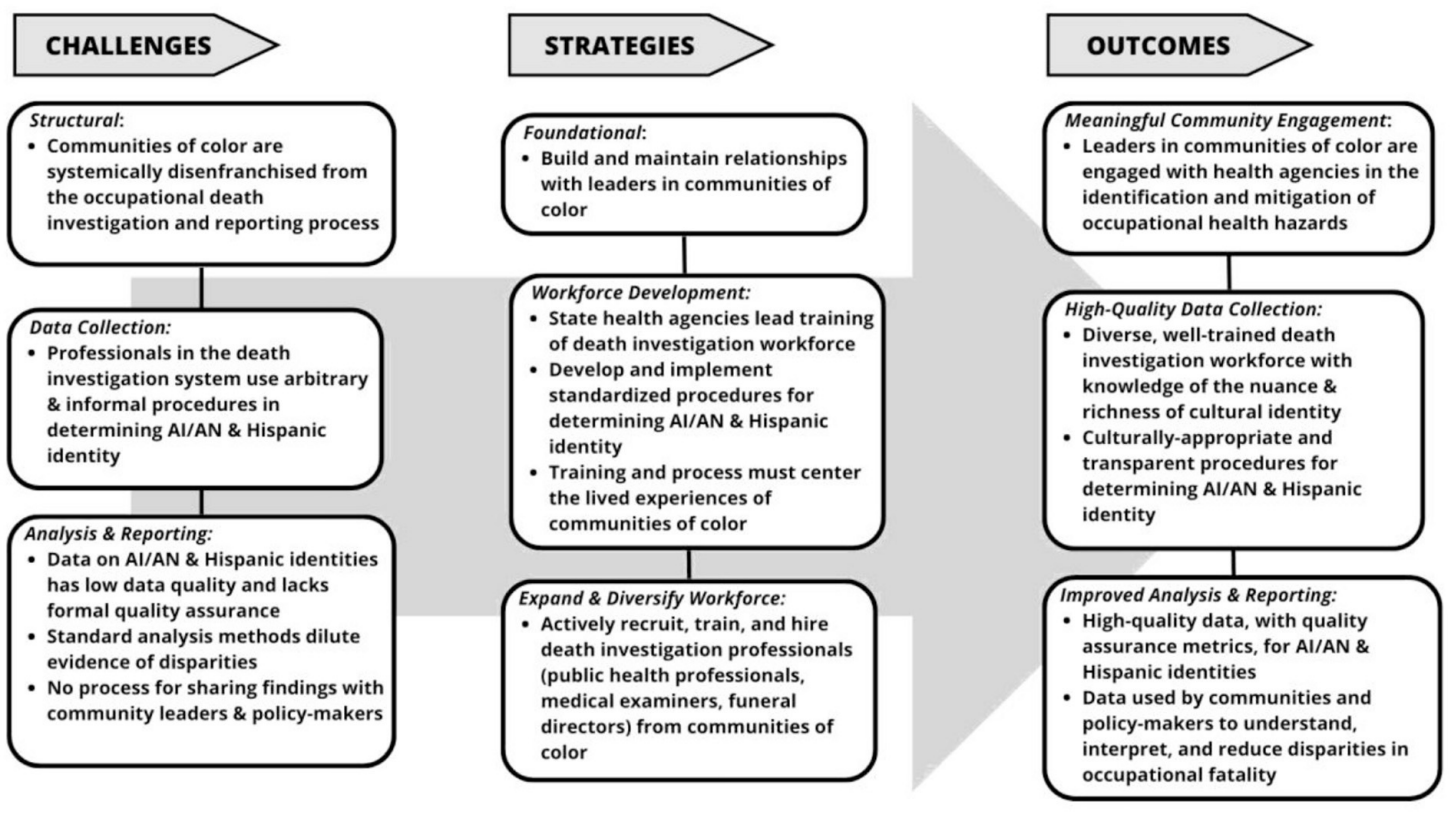
Challenges, strategies, and outcomes related to standard, accurate, and consistent reporting the race and ethnicity of occupational decedents.

In Figure 3, we summarize the challenges to accurate reporting and recording of race and ethnicity among decedents of occupational injuries, broken into three areas: structural, data collection, and analysis and reporting. We also provide recommended strategies for mitigating harm related to these challenges, again broken into three categories: foundational, workforce development, and expand workforce. Finally, we include possible positive outcomes of the recommendations in three areas: engagement, data collection process, and analysis and reporting.

## Conclusion

It is critically important to improve current methods, policies, and practices in the collection, analysis, and interpretation of data on American Indian/Alaska Native race and Hispanic ethnicity. Without substantial improvement in methodologies, we lack definitive information that is needed to quantify, monitor, and reduce health disparities, such as those in occupational fatalities in NC. Although this commentary focuses on misclassification of race/ethnicity in occupational fatalities, the concerns and recommendations we outline apply to many other sources of administrative health data. Researchers and consumers of research data should be mindful of the multiple sources of bias in data on race and ethnicity.

## Data availability statement

The original contributions presented in the study are included in the article/supplementary material, further inquiries can be directed to the corresponding author.

## Author contributions

EM conceptualized, drafted the initial manuscript, and created the figure. EM and DG conducted the literature review. DG, RB, and TC provided content knowledge on racial and ethnic identification in the context of funeral directors. MN assisted with the figure and misclassification analyses. SM and DR provided content knowledge on NC occupational fatality data collection processes and analysis history. All authors contributed to editing of the manuscript and approved the final manuscript as submitted.

## Funding

This study was supported by awards T42 OH008673 and R01 OH011256-01A1 from the National Institute for Occupational Safety and Health. The University of North Carolina Injury Prevention Research Center is partially supported by award R49/CE002479 from the National Center for Injury Prevention and Control, Centers for Disease Control and Prevention. Mortality data were provided by the Office of the Chief Medical Examiner, North Carolina Department of Health and Human Services.

## Conflict of interest

The authors declare that the research was conducted in the absence of any commercial or financial relationships that could be construed as a potential conflict of interest.

## Publisher's note

All claims expressed in this article are solely those of the authors and do not necessarily represent those of their affiliated organizations, or those of the publisher, the editors and the reviewers. Any product that may be evaluated in this article, or claim that may be made by its manufacturer, is not guaranteed or endorsed by the publisher.
